# What happens to migrant tuberculosis patients who are transferred out using a web-based system in China?

**DOI:** 10.1371/journal.pone.0206580

**Published:** 2018-11-08

**Authors:** Tao Li, Xin Du, Hemant Deepak Shewade, Kyaw Thu Soe, Hui Zhang

**Affiliations:** 1 National Center for Tuberculosis Control and Prevention, China CDC, Beijing, China; 2 International Union Against Tuberculosis and Lung Disease (The Union), South-East Asia Office, New Delhi, India; 3 International Union Against Tuberculosis and Lung Disease (The Union), Paris, France; 4 Department of Medical Research (Pyin Oo Lwin Branch), Ministry of Health and Sports, Pyin Oo Lwin, The Republic of The Union of Myanmar; Indian Institute of Technology Delhi, INDIA

## Abstract

**Background:**

In China, internal migrants constitute one-fifth of tuberculosis (TB) patients registered for treatment in web-based TB information management system (TBIMS). Though China added a specific module in the web-based TBIMS in 2009, web-based transfer-out is not specifically recommended in the national guidelines.

**Objective:**

In this country wide study among all registered migrant TB patients (2014–2015) that were transferred out using web-based TBIMS in China, to determine the i) timing of transfer-out in relation to period of treatment; ii) delay and attrition during transfer interval (between transfer-out and transfer-in); and iii) extent and risk factors for ‘not evaluated’ as the treatment outcome.

**Methods:**

This was a cohort study involving review of web-based TBIMS data. Modified Poisson regression was used to build a predictive model for risk factors of ‘not evaluated’ as the treatment outcome.

**Results:**

Among 7 284 patients, 5 900 (81.0%) were transferred out during the first two months after initiation of treatment or before treatment initiation and 7 088 (97.3%) patients had arrived at transfer-in unit. The median transfer interval was three (interquartile range: 0–14) days. Sixteen percent (1 176/7 284) patients had ‘not evaluated’ as their treatment outcome. ‘Not evaluated’ contributed to 66% of the unfavourable outcomes. Patients transferred from referral hospitals, migrated from out of prefecture, transferred out of prefecture, with sputum smear negative pulmonary TB, with TB pleurisy and with long delay between symptom onset and treatment initiation had significantly higher risk of ‘not evaluated’ as the outcome.

**Conclusion:**

Web-based transfer helped as the delay and attrition during the transfer interval was quite short and treatment outcomes of more than four-fifths of transferred out migrant TB patients were available with transfer-out BMU. Once strategies to address the independent predictors of ‘not evaluated’ treatment outcome are devised, China may consider mandatory use of web-based TBIMS for transferring out migrant TB patients.

## Introduction

Globally, tuberculosis (TB) is the ninth leading cause of death and the leading cause from a single infectious agent [1]. Despite the availability of highly effective anti-TB therapy, the treatment success rate for drug susceptible TB is 83% (less than 90% target) and 8–9% of patients’ treatment outcome is ‘not evaluated’–‘*a TB patient for whom no outcome is assigned’* [1–3]. Completeness of outcome reporting is an indicator of programme performance [1,4].

China runs a well-established, standardized recording and reporting system on patient finding and treatment outcomes. The TB Information Management System (TBIMS), a web-based information system, was built in 2005 by the National Center for TB Control and Prevention (NCTB). All designated hospitals, TB clinics and TB dispensaries were obligated to use this system to register confirmed patients. All TB patients managed by Nation TB Program (NTP) must be managed in TBIMS [5,6]. Nationally, the treatment success rate is 94% with 3–4% being ‘not evaluated’[1,4]. China is a high TB burden country with an estimated 895,000 patients annually, thus ‘not evaluated’ contributes to a significant number of patients [1]. Non-evaluation of treatment outcomes often happens among transferred out TB patients–meaning registered TB patients that are sent to other administrative units(called as transfer-in units) within the programme for treatment continuation any time during treatment [4].

**Fig 1:**
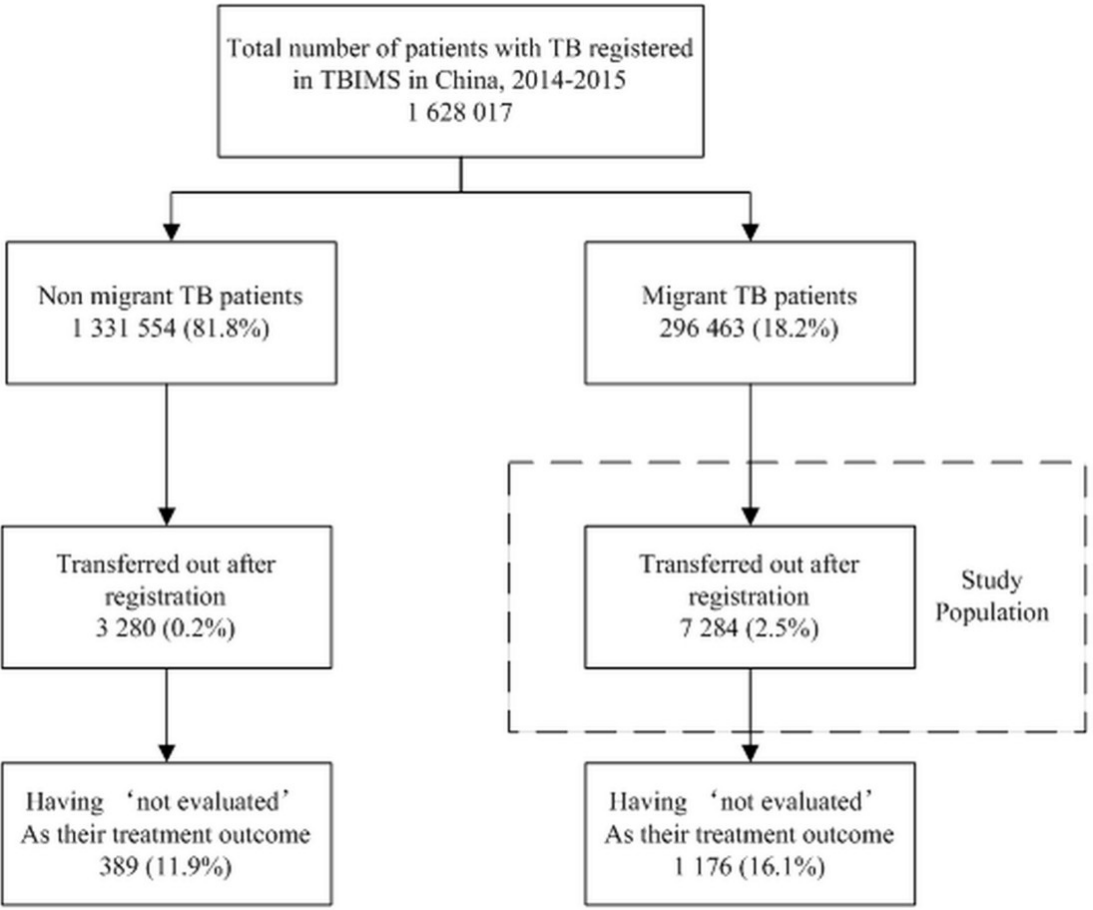
Flow chart depicting ‘not evaluated’ as the treatment outcome among migrant and non-migrant transferred out TB patients using TB information management system in China, 2014–2015. TBIMS–tuberculosis information management system; TB–tuberculosis.

In China, internal migrants (henceforth called as migrants) account for one-fifth of the whole population [7]. The estimated notification rate of active TB patients among migrants was 85 per 100 000 population (2014) when compared to overall estimated incidence of 64 per 100 000 population (2016) [1,8]. Between 2004 and 2015, there was an increase in TB notification rates among migrants while the national rates were decreasing [8]. In addition, the probability of being transferred out among migrants was quite higher than non-migrants (**Fig 1**).

Tuberculosis among cross-border migrants (immigrants) has been studied in detail [9–15]. There is dearth of knowledge on TB among internal migrants. In addition, there are limited published studies on how many transferred out TB patients have their treatment outcomes evaluated; especially among migrant TB patients [16].

In 2006, China took a policy decision to routinely capture migrant / non-migrant information and register migrant TB patients for treatment at their place of residence [17]. In 2009, to facilitate transfer out, China added a specific module in the web-based TBIMS [5]. This provides us a unique opportunity to study transferred-out migrant TB patients at country level. The use of web-based transfer out is not specifically recommended in the national guidelines [18]. This study has the potential to change or enhance China’s migrant TB strategies.

Hence, we conducted this country wide study among migrant TB patients that were transferred out using web-based TBIMS in China. Specific objectives were, to determine the i) timing of transfer-out in relation to period of treatment; ii) delay and attrition between transfer out and transfer in; and iii) extent and risk factors for ‘not evaluated’ as the treatment outcome.

## Methods

### Study design

This was a cohort study involving review of web-based TBIMS data.

### Setting

#### General setting

China has a population of over 1.4 billion and is the world's most populous country [1]. It has 3 levels of sub-national administrative division: 34 provinces or regions, 333 prefectures and more than 3000 counties.

The NCTB, which belongs to China center for disease control, is in charge of the NTP. TB management units are established at provincial, prefecture and county levels (basic management units (BMU) at county level). TB diagnostic facilities are centralized at the BMU level and rarely available at township level (below county). Diagnosed patients are registered in web-based TBIMS and initiated on directly observed therapy (DOT) at BMU with assistance from township clinics and village health workers. There are some regional referral hospitals which also take patient management responsibility similar to a BMU.

#### China web-based TBIMS

**Fig 2:**
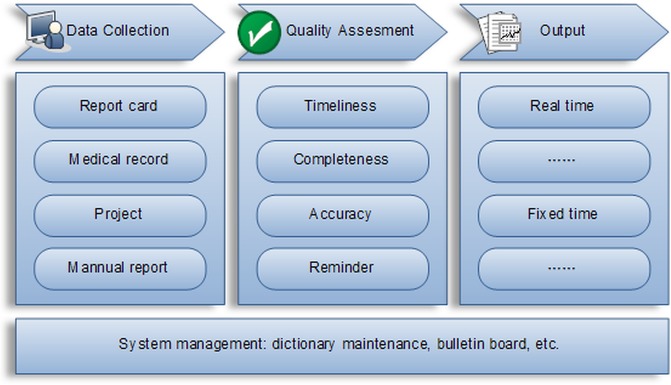
Diagram depicting functions of electronic TB information management system (TBIMS), China.

The function of web-based TBIMS can be divided into 4 categories: data collection, quality assessment, output and system management [6] (**Fig 2).** The transfer of registered TB patients happens along with all medical documents in electronic format. If the patient is traced successfully by the transfer-in BMUs, they take over patient’s management and update the outcome in web-based TBIMS when patients finish their treatment. This information can be visualized (with no rights to modify) by the transfer-out BMU [6]. If there is no outcome related information in the web-based TBIMS at the end of one year after date of registration, then these patients are reported as ‘not evaluated’ by the transfer-out BMUs. The transfer-in BMUs do not include them in their quarterly and annual reports.

### Study population and period

All migrant patients with TB registered in China during 2014–15 that were transferred out using web-based TBIMS anytime during their treatment were included in the study (**Fig 1**). Any patient who resided in the county for less than six months at the time of registration or belonged to another county was classified as ‘migrant’ in the web-based TBIMS.

### Data collection

Secondary data was extracted from web-based TBIMS in Microsoft Excel (Microsoft, Redmond, WA, USA). Socio-demographic (age, gender, occupation and permanent residence of patient), clinical (TB classification, treatment category and HIV status), health system (registration/transfer-out BMU name, transfer-in BMU name, dates of symptom onset, first doctor visit, diagnosis, registration, treatment initiation, transfer out and transfer in) variables and treatment outcomes were collected. Treatment outcomes, as per WHO recommendations, have been depicted in **Table 1** [4].

**Table 1:** Operational definition of TB treatment outcomes used in the study, China (2014–15)[4].

Outcome	Definition*
Cured	A pulmonary TB patient with bacteriologically confirmed TB at the beginning of treatment who was smear- or culture-negative in the last month of treatment and on at least one previous occasion.
Treatment completed	A TB patient who completed treatment without evidence of failure BUT with no record to show that sputum smear or culture results in the last month of treatment and on at least one previous occasion were negative, either because tests were not done or because results are unavailable.
Treatment failed	A TB patient whose sputum smear or culture is positive at month 5 or later during treatment.
Died	A TB patient who dies for any reason before starting or during the course of treatment.
Lost to follow-up	A TB patient who did not start treatment or whose treatment was interrupted for 2 consecutive months or more.
Not evaluated	A TB patient for whom no treatment outcome is assigned. This includes patients “transferred out” to another treatment unit as well as patients for whom the treatment outcome is unknown to the reporting unit.
Treatment success	The sum of cured and treatment completed.
Unfavourable outcome	All outcomes other than cured and treatment completed

*WHO: Definitions and reporting framework for tuberculosis– 2013 revision (updated December 2014)

### Data management and analysis

Database was constructed, cleaned and analyzed with Microsoft Excel (Microsoft, Redmond, WA, USA). Adjusted analysis was done using STATA (version 12.1, copyright 1985–2011 Stata Corp LP USA).

#### Derived variables

We followed similar definitions of delays as suggested by Sreeramareddy CT et al [19]. Patient delay was the time interval from onset of symptoms to first visit to a health facility. Diagnostic delay was from first visit to a health facility to confirmation of TB. Treatment delay was from confirmation of TB to treatment initiation. Health system delay comprised of diagnostic delay and treatment delay. Total delay comprised of patient delay and health system delay. Transfer from referral hospital was derived from registration BMU name. Type of transfer out (within prefecture / within province / out of province) was assigned by comparing the codes of transfer-out and transfers-in BMU. Timing of transfer out in relation to period of TB treatment was derived from dates of treatment initiation and transfer out. The transfer interval was calculated using dates of transfer out and transfer in.

#### Data analysis

Frequency (proportion) was used to summarize the timing of transfer out in relation to period of treatment (in months after treatment initiation); attrition between transfer out and transfer in; and extent of ‘not evaluated’ as the treatment outcome at the end of one year after registration. Median and interquartile range (IQR) were used to summarize delays/intervals.

Modified Poisson regression was used to build a predictive model (forward stepwise) for the risk factors associated with ‘not evaluated’ as the treatment outcome. Age, gender and variables with unadjusted p value <0.20 were considered in the model. Considering high multi-collinearity among all the delay variables, we decided to include only the ‘total delay’ variable. Age group, gender and TB category were not retained during model building. Unadjusted and adjusted RR (0.95 CI) was used to summarize (infer) the association of the variables included in the final model.

### Ethics approval

Ethics approval was obtained from the Ethics Advisory Group (EAG) of International Union Against Tuberculosis and Lung Disease (The Union), Paris, France (EAG No: 30/17) and Ethics Committee of Chinese Center for Disease Control and Prevention (Number: 201704). Administrative approval to conduct the study was sought from NCTB, China. As the study involving review of secondary data, waiver of written informed consent was sought and waived off by the ethics committees.

## Results

There were 1 628 017 patients with TB registered in web-based TBIMS during 2014–15. Among them, 296 463 (18.2%) patients were identified as migrants of whom 7 284 (2.5%) patients got transferred out using web-based TBIMS **(Fig 1).**

### Baseline characteristics

The characteristics of 7 284 study participants at registration have been summarized in **Table 2**.The mean (SD) age was 41.1 (17.9) years and 5 107 (70.1%) were males. Of the total, 1 859 (25.5%) had their permanent residence out of the province, 5 153 (57%) were transferred out by referral hospitals and 1 339 (18.4%) were transferred out of the province.

**Table 2:** Socio-demographic, clinical and health system related characteristics of migrant TB patients that were transferred out using web-based TBIMS, China (2014–2015).

Characteristics			N	(%)
Total			7284	(100.0)
**Socio-demographic characteristics**				
Age group				
	<15		33	(0.5)
	15–44		4261	(58.5)
	45–64		2126	(29.2)
	> = 65		864	(11.9)
Gender				
	Male		5107	(70.1)
	Female		2177	(29.9)
Occupation				
	Studying		492	(6.8)
	Farmers and herdsmen		2321	(31.9)
	Semi-skilled employee		107	(1.5)
	Salary employee		1294	(17.8)
	Non-salary employee		290	(4.0)
	Unemployed		2328	(32.0)
	Others		452	(6.2)
Residency*				
	Within prefecture		4871	(66.9)
	Within province		557	(7.6)
	Out of province		1856	(25.5)
**Clinical characteristics**				
Classification				
	Smear positive		2440	(33.5)
	Smear negative		4324	(59.4)
	PTB smear status unknown		34	(0.5)
	Pleurisy		483	(6.6)
	EPTB		3	(0.0)
Category				
	New		6915	(94.9)
	Retreated		369	(5.1)
HIV				
	Positive		9	(0.1)
	Negative		2864	(39.3)
	Unknown#		4411	(60.6)
**Health system related characteristics**				
Transferred from Referral hospital				
	Yes		4153	(57.0)
	No		3131	(43.0)
Type of transfer-out*				
	Within prefecture		4469	(61.4)
	Within province		1476	(20.3)
	Out of province		1339	(18.4)

Column percentages. TB–tuberculosis; TBIMS–tuberculosis information management system; PTB–pulmonary tuberculosis; EPTB–extrapulmonary tuberculosis; HIV–human immunodeficiency virus

*Residency–Within prefecture: patients came from another county but belonged to the same prefecture; within province: patients came from another county in different prefecture but from same province; out of province: patients came from another county belonging to different province

**#** TB examinations were routinely carried out in all new or follow up HIV/AIDS patients nationwide while TB patients were screened with HIV tests only in selected high HIV epidemic counties.

Delays before registration have been depicted in **Table 3.** The median total delay was 22 (IQR: 11–41) days. While median patient level delay was 16 (IQR: 6–34) days, health system level delay was 2 (IQR: 0, 6) days.

**Table 3:** Patient and health system delay in diagnosis and treatment initiation among migrant TB patients that were transferred out using web-based TBIMS, China (2014–2015) (N = 7284)*.

Delays	Patients	Median in days	(IQR)
Patient delay (a)	7275	16	(6–34)
Health system diagnosis delay (b)	7283	1	(0–5)
Health system treatment delay (c)	7270	0	(0–0)
Health system delay (b+c)	7269	2	(0–6)
Diagnosis delay (a+b)	7275	21	(10–40)
Total Delay (a+b+c)	7262	22	(11–41)

TB–tuberculosis; IQR–Interquartile Range; TBIMS–tuberculosis information management system

*dates missing for some patients

a—Patient delay–date of symptom onset to date of first visit to a doctor.

b -Health system diagnosis delay–date of first visit to a doctor to date of diagnosis as TB.

c—Health system treatment delay–date of diagnosis to date of treatment initiation.

### Transfer out in relation to period of treatment, transfer interval and attrition

Of 7 284, a total of 5 900 (81.0%) patients were transferred out during the first 2 months after initiation of treatment or before treatment start. A total of 7 088 (97.3%) patients arrived at transfer-in BMU. The median transfer interval was three (IQR: 0–14) days **(Table 4).**

**Table 4:** Transfer-out time and duration between transfer-out and transfer-in among migrant TB patients that were transferred out using web-based TBIMS, China (2014–2015).

Transfer-out time	N	(%)
Total	7284	(100.0)
Immediately after registration*	143	(2.0)
1st month after initiation of treatment	4162	(57.1)
2nd month after initiation of treatment	1595	(21.9)
3rd month after initiation of treatment	671	(9.2)
4th month after initiation of treatment	366	(5.0)
5th month after initiation of treatment	182	(2.5)
6th month and above after initiation of treatment	165	(2.3)
Number and percentage of transferred out migrant patients with TB arrived at destination BMU	7088	(97.3)
Median time for transfer-out	**Median in days**	**(IQR)**
	3	(0–14)

TB–tuberculosis; IQR–Interquartile Range; BMU–Basic Management Unit; TBIMS–tuberculosis information management system

*Immediately after registration: patients got transferred out immediately after registration and didn't start treatment in the transfer-out BMU.

### Treatment outcomes

Of 7284, 1 176(16.1%) patients had ‘not evaluated’ as their treatment outcome. A total of 1 785 (24.5%) had unfavourable outcomes which included ‘not evaluated’ as well **(Fig 1).** ‘Not evaluated’ contributed to 66% of the unfavourable outcomes **(Table 5).**

**Table 5:** Treatment outcomes of migrant TB patients that were transferred out using web-based TBIMS, China (2014–2015).

**Treatment outcomes**	**N**	**(%)**
Total	7284	(100.0)
Cured	1820	(25.1)
Treatment completed	3679	(50.6)
Failure	24	(0.3)
Died	44	(0.6)
Lost to follow up	375	(5.1)
Transferred to MDR cohort*	10	(0.1)
Others**	156	(2.1)
Not evaluated	1176	(16.1)

TB–tuberculosis; TBIMS–tuberculosis information management system; MDR–multidrug resistant TB cohort

*we don’t have information if these patients were started on second line treatment, hence retained in the cohort [4]

**There were some patients that didn’t match any of WHO outcome definition, e.g. patients got excluded as TB during treatment; these were recorded as others in China’s TBIMS.

### Risk factors for ‘not evaluated’ / unfavourable treatment outcome

Patients transferred from referral hospitals had 40% higher risk for ‘not evaluated’ as the outcome when compared to those referred from programme BMUs [aRR: 1.4 (0.95 CI: 1.2–1.6)]. Patients migrating from within province [aRR: 1.5 (0.95 CI: 1.2–1.8)] and out of province [aRR: 1.5 (0.95 CI: 1.3–1.8)] were more likely to be ‘not evaluated’ when compared to those migrating within prefecture. When compared to patients transferred out within prefecture, those transferred out within province [aRR: 2.0 (0.95 CI: 1.7–2.5)] and out of province [aRR: 2.4 (0.95 CI: 2.0–3.0)] had significantly higher risk. Long delay before registration was associated with high risk and sputum smear positive pulmonary TB was associated with low risk for ‘not evaluated’ as the outcome. Timing of transfer-out was not an independent predictor **(Table 6).**

**Table 6:** Risk factors associated with ‘not evaluated’ outcome among migrant TB patients that were transferred out using web-based TBIMS, China (2014–2015).

Factors		Total	Not evaluated N (%)	RR	aRR^^
		N		(95%CI)	(95%CI)
		7284	1176(16.1)		
Age group					
	<15	33	5(15.2)	1.3(0.6–3.1)	-^*#*^
	15–44	4261	775(18.2)	1.6(1.3–2.0)^	
	45–64	2126	299(14.1)	1.3(1.0–1.6)^	
	> = 65	864	97(11.2)	ref	
Gender					
	Male	5107	811(15.9)	ref	-^*#*^
	Female	2177	365(16.8)	1.1(0.9–1.2)	
Occupation					
	Studying	492	113(23.0)	2.6(2.1–3.1)^	2.3(1.9–2.7)^
	Farmers and herdsmen	2321	461(19.9)	2.2(1.9–2.6)^	2.3(1.9–2.6)^
	Semi-skilled employee	107	14(13.1)	1.5(0.9–2.4)	0.9(0.6–1.6)
	Salary employee	1294	226(17.5)	1.9(1.6–2.3)^	1.3(1.1–1.5)^
	Non-salary employee	290	66(22.8)	2.5(2.0–3.2)^	1.5(1.1–1.9)^
	Unemployed	2328	209(9.0)	ref	ref
	Others	452	87(19.2)	2.1(1.7–2.7)^	1.5(1.2–1.9)^
Residency*					
	Within prefecture	4871	611(12.5)	ref	ref
	Within province	557	108(19.4)	1.5(1.3–1.9)^	1.5(1.2–1.8)^
	Out of province	1856	457(24.6)	2.0(1.8–2.2)^	1.5(1.3–1.8)^
Classification					
	Smear positive	2440	303(12.4)	ref	ref
	Smear negative	4324	771(17.8)	1.4(1.3–1.6)^	1.4(1.2–1.6)^
	PTB smear status unknown	34	14(41.2)	3.3(2.2–5.0)^	2.5(1.6–3.9)^
	Pleurisy	483	86(17.8)	1.4(1.2–1.8)^	1.8(1.5–2.3)^
	EPTB	3	2(66.7)	5.4(2.4–12.0)^	8.7(3.3–22.9)^
Category					
	New	6915	1112(16.1)	ref	-^*#*^
	Retreated	369	64(17.3)	1.1(0.9–1.4)	
HIV					
	Positive	9	2(22.2)	2.3(0.7–8.0)	1.8(0.5–6.7)
	Negative	2864	272(9.5)	ref	Ref
	Unknown	4411	902(20.4)	2.2(1.9–2.4)^	2.0(1.8–2.3)^
Transferred from Referral hospital					
	Yes	4153	609(14.7)	0.8(0.7–0.9)	1.4(1.2–1.6)^
	No	3131	567(18.1)	ref	ref
Type of transfer					
	Within prefecture	4469	514(11.5)	ref	ref
	Within province	1476	316(21.4)	1.9(1.6–2.1)^	2.0(1.7–2.5)^
	Out of province	1339	346(25.8)	2.2(2.0–2.5)^	2.4(2.0–3.0)^
When the transfer happened					
	Immediately after registration**	4162	585(14.1)	ref	ref
	1st month after initiation of treatment	1593	329(20.7)	1.0(0.7–1.5)	0.9(0.6–1.2)
	2nd month after initiation of treatment	1386	242(17.5)	1.5(1.0–2.2)	1.1(0.8–1.5)
	3rd month and above after initiation of treatment	143	20(14.0)	1.2(0.8–1.9)	1.0(0.7–1.4)
Total Delay				1.0(1.0–1.0)	1.0(1.0–1.0)^

TB–tuberculosis; PTB–pulmonary tuberculosis; EPTB–extrapulmonary tuberculosis; RR–relative risk; aRR–adjusted relative risk; HIV–human immunodeficiency virus; TBIMS–tuberculosis information management system

*residency–within prefecture: patients came from another county but belonged to the same prefecture; within province: patients came from another county in different prefecture but from same province; out of province: patients came from another county belonging to different province

** patients got transferred out immediately after registration and didn't start treatment in the transfer-out BMU

^statistically significant

^^adjusted analysis using Modified Poisson regression with robust variance estimates (stepwise forward method), only total delay was considered for model building because of high multicollinearity among various types of delays. aRR for total delay = 1.0008 (0.95 CI: 1.0005–1.0011)

^#^age group”, gender” and “TB category” were not retained in final model.

Predictors for unfavourable outcome were more or less similar to predictors for ‘not evaluated’ **(S1 Table).**

## Discussion

This is the first ever study globally to assess treatment outcomes and risk factors of non-evaluation among transferred out migrant TB patients and that too involving a web-based system. As one of the most important subgroups of vulnerable populations, TB control in migrants was always considered as a high priority in WHO Stop TB and End TB strategies [20,21]. Tracking and managing transferred-out migrant TB patients could be tricky. In this background, our study had some key findings.

The use of web-based transfer mechanism in China helped the programme to manage transferred-out migrant TB patients. The delay and attrition during the transfer interval was minimal and treatment outcomes of more than four-fifths of transferred-out migrant TB patients were evaluated and available with transfer-out BMU. In Zimbabwe (2010), the median transfer interval was eight days and treatment outcomes were available with transfer-out unit in less than two percent of patients [16]. In Ethiopia (2009–13), treatment outcome for none of the transferred in patients was communicated back to transfer-out unit [22]. Both these studies were in settings with paper-based transfer-outs.

China started to focus on the migrant TB problem from 2006 [17], but before 2009, the transfer-out BMU gave paper-based transfer forms and the patients had to contact the transfer-in BMU themselves. Anecdotally, patients rarely reached the transfer-in BMU to receive treatment so that treatment outcomes were difficult to evaluate. Moreover, there was also the lack of coordination mechanism among BMUs which was addressed by the web-based TBIMS [23,24]. The TBIMS recorded patients’ contact information like mobile phone number (including those of relative, if available) and address of transfer-out/in place [5,6].

Around three-fifths of migrant patients with TB were transferred out within 1 month of treatment initiation. The major reasons for migrant patients being transferred out could be interruption of current job or study related [25]. These actions may mostly happen shortly after diagnosis. On the other hand, when a patient has already got a relevant long period of management at a certain place, their willingness for transfer out could pass away.

The proportion of unfavourable outcomes among transferred-out migrant TB patients was 23% for sputum smear positive pulmonary TB and 29% for previously treated TB patients. These figures for migrant TB patients in general (not transferred out) were 8% and 18% respectively [26]. Therefore, among migrant TB, the treatment outcomes were worse if the patient was transferred out. This difference appears to be due to high ‘non-evaluated’ outcome (two-thirds of unfavourable outcomes) among transferred-out migrant TB patients. Similar findings were found elsewhere but among non-migrant patients. Unfavourable outcome and ‘not evaluated’ outcome among transferred in patients was 31% and 21%, respectively, in Zimbabwe (2010) and 21% and 14%, respectively, in Ethiopia (2009–13) [16,22].

High risk for ‘not evaluated’ outcome among patients who migrated from a far distant place and among migrant patients who were transferred out to a far distant place was indicative of the challenges of coordination among BMUs in different provinces. This has been documented previously as well [27,28]. We speculate that for patients transferred out beyond a prefecture, because of a possible lack of personal contact among the BMU nodal persons, the transfer-out BMU may be completely dependent on the nodal person contact details in web-based TBIMS. If these are not updated, it might become difficult to contact the BMU and track the patient.

Patient being transferred out from a referral hospital was an independent predictor for ‘not evaluated’ outcome. Though the unadjusted RR indicated a protective effect, after adjusting for other potential factors there was a reversal of effect. More than half of the study population was from referral hospitals; hence this factor is very important. Lack of knowledge or willingness to instruct patients on importance of standardized full course anti-tuberculosis treatment among doctors of referral hospital has been documented [29,30]. In addition, lack of coordination between referral hospital and programme may also contribute to ‘not evaluated’ outcome [29].

Smear positive patients were less likely to have ‘not evaluated’ as their treatment outcome, this could result from NTP paying more attention to these patients with high transmission potential. They may also have more severe symptoms which may be the incentive to continue treatment.

Analysis also indicated that risks of ‘not evaluated’ outcome increased when patients had long delay before initiation of treatment. It is possible that several unmeasured patient level characteristics like income, knowledge, attitude and belief systems which contributed to delay also continued to contribute to ‘not evaluated’ outcome.

### Implications for policy and practice

The findings of this study have been submitted to NCTB. It has been decided to add a precise instruction in the upcoming revised national TB guidelines (last national guideline was in 2009) regarding the mandatory use of web-based TBIMS for transfer-out of all migrant TB in China. This study provides evidence base for other high TB burden countries as well to develop a web-based TBIMS in line with WHO recommendations [5].

Despite most of the patients reaching the transfer-in BMU (97%), outcomes were only available for 84% of patients. This gap of 13% indicates that outcomes for many patients were not evaluated even after arrival in transfer-in BMU. We have identified certain important predictors. There is an urgent need to ensure mechanisms to improve the coordination between referral hospitals and programme BMUs so that patients transferred from referral hospitals are tracked, managed and their outcomes updated in TBIMS. To address the issue of high ‘not evaluated’ outcome if the transfer out is out of prefecture, the programme may consider regular updating of the BMU nodal person contact details in web-based TBIMS. However, a systematic qualitative enquiry is required to know the exact programmatic and patient level perspectives and reasons.

### Strengths and limitations

Globally, few countries collect sufficiently disaggregated data on the health of migrants [31,32]. This is a major strength of the study. Under sustainable development goals (SDG), SDG 17 includes two targets and associated indicators under the subheading of “data, monitoring and accountability” which include mechanism to generate disaggregate data for specific subpopulations. Migrants are one such subpopulation [1,33]. Second, the study involved a large cohort of patients and the findings are representative of the situation in China. Third, despite being a record review, there were minimal missing values.

There were two limitations. First, being a record review of programme data, we did not have information of other patient level characteristics such as smoking, alcohol use, family income, nutritional status, patient knowledge and attitude; and programmatic characteristics such as availability of staff and training status. Second, the field in web-based TBIMS providing reasons for non-evaluation was not a mandatory field, unlike other variables included in our study. Hence, this information was not filled for majority of the ‘not evaluated’ patients and we had to drop the objective on programme reported reasons for ‘not evaluated’ outcome. We speculate it was also caused by migrants’ instability and many of these ‘not evaluated’ patients were actually lost to follow-up. To get further information, we need to update our TBIMS or do more qualitative studies.

## Conclusion

The use of web-based TBIMS as a transfer mechanism in China helped the programme to manage transferred out migrant TB patients very well. This strategy could be considered as a recommendation in future guideline and implemented nationwide systematically. Some important predictors for ‘not evaluated’ were identified and the programme should devise strategies to address these. For those ‘not evaluated’ transferred-out migrant patients, we need to carry out further studies to further understand the reasons and improve the results.

## Supporting information

S1 Annex STATA dataset file for analysis. (RAR)

S1 Table Risk factors associated with unfavorable outcomes among migrant TB patients that were transferred out using web-based TBIMS, China (2014–2015). (DOCX)

## References

[1] World Health Organization (WHO). Global tuberculosis report 2017. WHO/HTM/TB/2017.23.Geneva, Switzerland; 2017.

[2] United Nations Office for Project Services. The paradigm shift 2016–2020, Global Plan to End TB. Geneva Switzerland; 2015.

[3] World Health Organization (WHO). Guideline for drug suspectible tuberculosis and patient care. WHO/HTM/TB/2017.05.Geneve,Switzerland; 2017.

[4] World Health Organization (WHO). Definitions and Reporting Framework for Tuberculosis- 2013 revision (updated December 2014). Geneva; 2014.

[5] World Health Organization (WHO). Electronic recording and reporting for tuberculosis care and control. WHO/HTM/TB/2011.22.Geneve,Switzerland; 2012.

[6] Huang F, Cheng S, Du X, Chen W, Scano F, Falzon D, et al Electronic recording and reporting system for tuberculosis in China: experience and opportunities. J Am Med Inform Assoc. 2014;21: 938–41. 10.1136/amiajnl-2013-002001 24326537 PMC4147602

[7] China Statistics Press. China statistical yearbook 2015 [Internet]. 2016 [cited 15 Nov 2017]. Available: http://www.stats.gov.cn/tjsj/ndsj/2015/indexch.htm

[8] Sun Y-X, Zhu L, Lu Z-H, Jia Z-W. Notification Rate of Tuberculosis among Migrants in China 2005–2014: A Systematic Review and Meta-analysis. Chin Med J (Engl). 2016;129: 1856 10.4103/0366-6999.186650 27453237 PMC4976576

[9] Arshad S, Bavan L, Gajari K, Paget SNJ, Baussano I. Active screening at entry for tuberculosis among new immigrants: A systematic review and meta-analysis. Eur Respir J. 2010;35: 1336–1345. 10.1183/09031936.00054709 19840970

[10] Dara M, Sulis G, Centis R, D’Ambrosio L, de Vries G, Douglas P, et al Cross-border collaboration for improved tuberculosis prevention and care: policies, tools and experiences. Int J Tuberc Lung Dis. 2017;21: 727–736. 10.5588/ijtld.16.0940 28633696

[11] White PJ, Abubakar I, Aldridge RW, Hayward AC. Post-migration follow-up of migrants at risk of tuberculosis. Lancet Infect Dis. Elsevier Ltd; 2017;17: 1124 10.1016/S1473-3099(17)30567-4 29115264

[12] Chan IHY, Kaushik N, Dobler CC. Post-migration follow-up of migrants identified to be at increased risk of developing tuberculosis at pre-migration screening: a systematic review and meta-analysis. Lancet Infect Dis. 2017;17: 770–779. 10.1016/S1473-3099(17)30194-9 28410979

[13] Abarca Tomás B, Pell C, Bueno Cavanillas A, Guillén Solvas J, Pool R, Roura M. Tuberculosis in migrant populations. A systematic review of the qualitative literature. PLoS One. 2013;8: e82440 10.1371/journal.pone.0082440 24349284 PMC3857814

[14] Scotto G, Fazio V, Muzio L Lo. Tuberculosis in the immigrant population in Italy: State-of-the-art review. Infez Med. 2017;25: 199–209. 28956536

[15] Bozorgmehr K, Razum O, Saure D, Joggerst B, Szecsenyi J, Stock C. Yield of active screening for tuberculosis among asylum seekers in Germany: A systematic review and meta-analysis. Eurosurveillance. 2017;22 10.2807/1560-7917.ES.2017.22.12.30491 28367795 PMC5388130

[16] Takarinda KC, Harries AD, Mutasa-Apollo T, Sandy C, Mugurungi O. Characteristics and treatment outcomes of tuberculosis patients who “transfer-in” to health facilities in Harare City, Zimbabwe: a descriptive cross-sectional study. BMC Public Health. BMC Public Health; 2012;12: 981 10.1186/1471-2458-12-981 23150928 PMC3585460

[17] Chinese Center for Diseases Control and Prevention. The Global Fund Tuberculosis Program in China: achivements and experiences. Beijing,China; 2015.

[18] Ministry of Health. Guidelines for Implementing the National Tuberculosis Control Program in China (2008). Beijing,China; 2009.

[19] Sreeramareddy CT, Qin ZZ, Satyanarayana S, Subbaraman R, Pai M, Zhen Qin Z, et al Delays in diagnosis and treatment of pulmonary tuberculosis in India: a systematic review. Int J Tuberc Lung Dis. 2014;18: 255–266. 10.5588/ijtld.13.0585 24670558 PMC4070850

[20] World Health Organization. The Stop TB Strategy: building on and enhancing DOTS to meet the TB-related Millennium Development Goals. 2006.

[21] World Health Organization (WHO). Implementing the End TB Strategy: The Essentials. WHO/HTM/TB/2015.31.Geneva, Switzerland; 2015.

[22] Belayneh T, Kassu A, Tigabu D, Asmare G, Tilaye S, Klinkenberg E. Characteristics and Treatment Outcomes of “Transfer-Out” Pulmonary Tuberculosis Patients in Gondar, Ethiopia. Tuberc Res Treat. 2016; 1 10.1155/2016/1294876 PMC4904106 27313887

[23] Ge L, Xueshan F, Shaokang Z. Epidemic and control strategies for migrant TB patients in China. Chinese J Public Heal. 2007;23: 701–3.

[24] Li X, Jiang S. Current situation and countermeasures for tuberculosis control among migrant population in China. Chin J Antituberc. 2009;31: 561–3.

[25] Jie L, Lianxin H, Qinlin C. Analysis on transfer cases during treatment in floationg population of tuberculosis patients and their management results in Hangzhou Economic and Technological Development Area(HEDA). Prev Med Trib. 2011;17: 438–440.

[26] Jiang S, Liu X. Progress and prospect of tuberculosis control for the floating population in China. Chin J Antituberc. 2014;36: 798–801.

[27] Zhou Y, Zhu L, Zhu Y, Xu W, Peng H, Xu X, et al Analysis on the implementation status of cross-regional management procedures and treatment outcome of the trans-provincial floating of TB cases. Chinese J Dis Control Prev. 2012;16: 874–6.

[28] Lin S, Dai Z, Wei S, Du Y. Effectiveness of treatment and management of pulmonary tuberculosis cases among cross-regional floating population. China Trop Med. 2011;11: 1334–1336.

[29] Liu X, Wang L, Du Y, Zhang H, Jiang S. Current status of case referring and treatment of tuberculosis impatients in four tuberculosis specialist hospitals in China. Chin J Antituberc. 2010;32: 775–8.

[30] Kiwuwa MS, Charles K, Harriet MK. Patient and health service delay in pulmonary tuberculosis patients attending a referral hospital: a cross-sectional study. BMC Public Health. 2005;5: 122 10.1186/1471-2458-5-122 16307685 PMC1310609

[31] World Health Organization Regional Office for the Western Pacific. Tuberculosis Control in Migrant Populations: Guiding Principles and Proposed Actions. Manila, Philippines; 2016.

[32] World Health Organization Executive Board. Health of migrants Migration flows and the globalized world. Geneva Switzerland; 2007.

[33] World Health Organization. Health in 2015: from MDGs, Millennium Development Goals to SDGs, Sustainable Development Goals. Geneva Switzerland; 2015.

