# Supplementary material for: What happens to migrant tuberculosis patients who are transferred out using a web-based system in China?
Source: PLoS One. 2018 Nov 8;13(11):e0206580. doi: 10.1371/journal.pone.0206580 (PMC6224056; doi:10.1371/journal.pone.0206580)
Supplement: S1 Table — (DOCX) [file pone.0206580.s002.docx]

**S2 Table. Risk factors associated with unfavorable outcomes among migrant TB patients that were transferred out using web-based TBIMS, China (2014-2015)**

| **Factors** | | **Total** | **Unfavorable outcomes**  **N (%)** | **RR** | **aRR^^** |
| --- | --- | --- | --- | --- | --- |
|  |  | **N** |  | **(95%CI)** | **(95%CI)** |
|  |  | 7284 | 1785(24.5) |  |  |
| Age group | |  |  |  |  |
|  | <15 | 33 | 10(30.3) | 1.6(0.9-2.7) | -*^#^* |
|  | 15-44 | 4261 | 1146(26.9) | 1.4(1.2-1.6)^ |  |
|  | 45-64 | 2126 | 461(21.7) | 1.1(1.0-1.3) |  |
|  | >=65 | 864 | 168(19.4) | ref |  |
| Gender | |  |  |  |  |
|  | Male | 5107 | 1269(24.8) | ref | -*^#^* |
|  | Female | 2177 | 516(23.7) | 1.0(0.9-1.0) |  |
| Occupation | |  |  |  |  |
|  | Studying | 492 | 143(29.1) | 1.6(1.3-1.8)^ | 1.4(1.2-1.6)^ |
|  | Farmers and herdsmen | 2321 | 563(24.3) | 1.3(1.2-1.4)^ | 1.4(1.3-1.6)^ |
|  | Semi-skilled employee | 107 | 22(20.6) | 1.1(0.7-1.6) | 0.8(0.5-1.1) |
|  | Salary employee | 1294 | 377(29.1) | 1.6(1.4-1.8)^ | 1.1(1.0-1.3) |
|  | Non-salary employee | 290 | 99(34.1) | 1.8(1.5-2.2)^ | 1.1(0.9-1.3) |
|  | Unemployed | 2328 | 436(18.7) | ref | ref |
|  | Others | 452 | 145(32.1) | 1.7(1.5-2.0)^ | 1.3(1.1-1.5)^ |
| Residency* | |  |  |  |  |
|  | Within prefecture | 4871 | 944(19.4) | ref | -*^#^* |
|  | Within province | 557 | 152(27.3) | 1.4(1.2-1.6)^ |  |
|  | Out of province | 1856 | 689(37.1) | 1.9(1.8-2.1)^ |  |
| Classification | |  |  |  |  |
|  | Smear positive | 2440 | 555(22.7) | ref | ref |
|  | Smear negative | 4324 | 1094(25.3) | 1.1(1.0-1.2)^ | 1.2(1.1-1.3)^ |
|  | PTB smear status unknown | 34 | 17(50.0) | 2.2(1.6-3.1)^ | 2.1(1.5-3.0)^ |
|  | Pleurisy | 483 | 117(24.2) | 1.1(0.9-1.3) | 1.5(1.3-1.8)^ |
|  | EPTB | 3 | 2(66.7) | 2.9(1.3-6.5)^ | 5.0(2.0-12.2)^ |
| Category | |  |  |  |  |
|  | New | 6915 | 1677(24.3) | ref | ref |
|  | Retreated | 369 | 108(29.3) | 1.2(1.0-1.4)^ | 1.5(1.3-1.8)^ |
| HIV | |  |  |  |  |
|  | Positive | 9 | 4(44.4) | 2.2(1.1-4.7)^ | 1.8(0.8-4.2) |
|  | Negative | 2864 | 570(19.9) | ref | ref |
|  | Unknown | 4411 | 1211(27.5) | 1.4(1.3-1.5)^ | 1.3(1.2-1.5)^ |
| Transferred from Referral hospital | |  |  |  |  |
|  | Yes | 4153 | 905(21.8) | 0.8(0.7-0.8) | 1.3(1.2-1.4)^ |
|  | No | 3131 | 880(28.1) | ref | ref |
| Type of transfer | |  |  |  |  |
|  | Within prefecture | 4469 | 733(16.4) | ref | ref |
|  | Within province | 1476 | 506(34.3) | 2.1(1.9-2.3)^ | 2.6(2.4-3.0)^ |
|  | Out of province | 1339 | 546(40.8) | 2.5(2.3-2.7)^ | 3.2(2.8-3.6)^ |
| When the transfer happened | |  |  |  |  |
|  | Immediately after registration** | 4162 | 930(22.3) | ref | -*^#^* |
|  | 1st month after initiation of treatment | 1593 | 440(27.6)) | 1.4(1.0-2.0) |  |
|  | 2nd month after initiation of treatment | 1386 | 392(28.3) | 1.7(1.2-2.5)^ |  |
|  | 3rd month and above | 143 | 23(16.1) | 1.8(1.2-2.6)^ |  |
| Total Delay^^ | |  |  | 1.0(1.0-1.0) | 1.0(1.0-1.0)^ |

*TB – tuberculosis; PTB – pulmonary tuberculosis; EPTB – extrapulmonary tuberculosis; RR – relative risk; aRR – adjusted relative risk; HIV – human immunodeficiency virus; TBIMS – tuberculosis information management system*

**residency– within prefecture: patients came from another county but belonged to the same prefecture; within province: patients came from another county in different prefecture but from same province; out of province: patients came from another county belonging to different province*

*** patients got transferred out immediately after registration and didn't start treatment in the transfer-out BMU*

*^statistically significant*

*^^adjusted analysis using Modified Poisson regression with robust variance estimates (stepwise forward method), only total delay was considered for model building because of high multicollinearity among various types of delays. aRR for total delaty = 1.0004 (0.95 CI: 1.0002-1.0007)*

*# age group, gender, residency and when the transfer happened were not retained in final model.*
